# Effects of COVID‐19 on Corneal Transplants Across the Five Regions of Brazil and Trends in Post‐Pandemic Recovery—A Cross‐Sectional Study

**DOI:** 10.1002/hsr2.71813

**Published:** 2026-02-17

**Authors:** Josue Geraldo Lessa, Amélia Soares de Melo, Leandro Vassuler Baldon, Bernardo G. L. Carvalho, Nathalia Sernizon Guimaraes, Aleida N. Soares, Bruno Lovaglio Cançado Trindade, Fernanda Ludolf

**Affiliations:** ^1^ Mestrado em Ciências da Saúde, Faculdade Ciências Médicas de Minas Gerais (FCMMG) Belo Horizonte Minas Gerais Brazil; ^2^ Instituto de Olhos Ciências Médicas (IOCM), Faculdade Ciências Médicas de Minas Gerais (FCMMG) Belo Horizonte Minas Gerais Brazil; ^3^ Medical School, Faculdade Ciências Médicas de Minas Gerais (FCMMG) Belo Horizonte Minas Gerais Brazil; ^4^ Departamento de Nutrição, Escola de Enfermagem Universidade Federal de Minas Gerais Belo Horizonte Minas Gerais Brazil; ^5^ Faculdade de Saúde Santa Casa Belo Horizonte Minas Gerais Brazil; ^6^ Programa de Pós‐Graduação em Ciências da Saúde: Infectologia e Medicina Tropical Universidade Federal de Minas Gerais Belo Horizonte Minas Gerais Brazil

**Keywords:** Brazil, cornea transplantation, COVID‐19, pandemia, recovery, SARS‐CoV‐2

## Abstract

**Background and Aims:**

The COVID‐19 pandemic significantly disrupted global eye health services, including corneal transplants. This study evaluates the pandemic's long‐term impact on corneal transplantation across Brazil's five regions, highlighting national and regional recovery trends.

**Method:**

Monthly corneal transplantation data were obtained from the Brazilian Health Information System (DATASUS) and analyzed across four periods: pre‐pandemic (January 2018 to February 2020), lockdown (March 2020 to December 2020), vaccination (January 2021 to March 2022), and post‐pandemic (April 2022 to June 2024) across the five Brazilian regions.

**Results:**

Transplants dropped by 54% in 2020, with the South, Northeast, and Southeast most affected. In 2021, as vaccination efforts advanced, transplants recovered by 86%, particularly in the South, Northeast, and Southeast. The upward trend continued in 2022, with an 18% increase nationwide, especially in the North and South. However, in 2023, there was a slight overall decrease of 5%, with declines in the North, Southeast, and Midwest, while the South and Northeast saw minor increases.

**Conclusion:**

Corneal transplantation in Brazil has shown signs of recovery, but a backlog remains, especially in underserved regions. This study underscores the need for continued monitoring and policy adjustments to address post‐pandemic demand and promote equitable access to eye care.

## Summary

- Corneal transplants in Brazil fell 54% during the 2020 pandemic lockdown.
- Recovery was strongest in South, Southeast, and Northeast after vaccination.
- Regional disparities persisted despite the overall transplant rebound by 2022.
- Findings call for targeted policies to ensure equitable post‐COVID‐19 eye care.

## 1 Introduction

In March 2020, the World Health Organization (WHO) declared Coronavirus Disease 2019 (COVID‐19) a pandemic, after Severe Acute Respiratory Syndrome Coronavirus 2 (SARS‐CoV‐2) spread rapidly, causing a high mortality rate worldwide [1, 2]. In Brazil, strict lockdown measures were fast implemented as early as March 2020 [3]. Just as distancing measures were implemented, flexibilization measures were adopted at varying levels by each Brazilian state, according to their specific conditions [4, 5]. In December 2020, driven by a new wave of infections a very significant increase in mortality rate occurred, however, more stringent isolation measures, like those at the start of the pandemic, were not reintroduced [3]. Only from March 2021 onward was Brazil able to secure a sufficient supply of vaccines and a year after, on April 2022, the Brazilian Ministry of Health declared the end of the Public Health Emergency of National Importance due to COVID‐19 [6, 7, 8, 9]. A year later, in May 2023, the WHO declared the end of the Public Health Emergency of International Concern regarding COVID‐19 [10].

During the COVID‐19 pandemic, healthcare systems worldwide reorganized to prioritize COVID‐19 care, reallocating resources and suspending elective appointments and surgeries to manage the surge in demand [11]. In this context, ophthalmological societies, including the American Academy of Ophthalmology (AAO), the American Society of Retina Specialists (ASRS), and the Brazilian Council of Ophthalmology (CBO), provided guidelines for classifying surgical procedures as urgent and non‐urgent aiming to contain the spread of the disease and conserve resources. They recommended the immediate suspension of any non‐urgent procedure starting in March 2020, for a minimum of 4 weeks, which ultimately extended until July 2020 [12, 13, 14].

Corneal transplantation is the most performed allogeneic transplant in the world [15, 16]. It can be used to treat multiple conditions that can ultimately lead to blindness, such as keratoconus, corneal opacities or scars, corneal dystrophies, and bullous keratopathy, among many others [17]. In some cases, urgent surgery may be required to restore ocular integrity (such as in trauma) or to eliminate an infectious keratitis [15, 17, 18].

The exceeding demand over supply of corneal tissue is a concern in many regions of the world. Prior to the COVID‐19 pandemic, a significant imbalance existed between the supply and demand for corneal tissue with an estimate that over 53% of the global population lacked access to corneal tissue, with only one available cornea for every 70 needed [15]. This disparity is likely to have worsened during the pandemic as seen around the world [19, 20, 21, 22, 23, 24].

The impacts on corneal donations and transplants in Brazil were observed from the outset of the pandemic, when the Ministry of Health imposed restrictions on eye tissue donations for nearly 6 months, between April and September 2020. Among these restrictions, it was established that cornea tissue could only be obtained from deceased multi‐organ donors with brain death, who showed no clinical or epidemiological signs of COVID‐19, and whose SARS‐CoV‐2 infection status was confirmed by a negative RT‐PCR test, with the sample collected 24 h before the removal of the eye [25].

There was a perception that ocular health had declined at the population level during the Covid‐19 pandemic, with a decrease in ocular transplant procedures, which can be due to the decrease in the number of cornea donations, available professionals, and government health investment relocation. The return of the number of corneal transplants, with the recovery of the suppressed demand, was expected in the years following the pandemic. However, the literature lacks studies that support these perceptions in Brazil, which is a vast country with notable regional disparities, making it even more important to understand the broader context. It is therefore crucial to collect and provide an objective analysis of data to investigate the pandemic's trends and recovery on ophthalmic services in Brazil, with the goal of informing policies and practices that can mitigate these negative effects and ensure equitable access to eye care.

## 2 Material and Methods

### 2.1 Ethics and Reporting Statement

As the data were extracted from an open‐access database, informed consent and ethical review were not required according to local ethical guidelines. This is a retrospective cross‐sectional study reported in accordance with the Strengthening the Reporting of Observational Studies in Epidemiology (STROBE) guidelines (https://www.equator-network.org/reporting-guidelines/strobe/) [26].

### 2.2 Data Analysis

**Figure 1:**
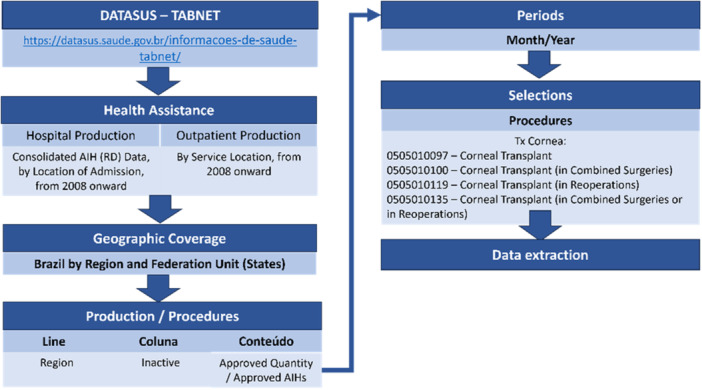
Data extraction flowchart.

Data were extracted from the Brazilian Health Information System website TABNET–DATASUS (https://datasus.saude.gov.br/informacoes-de-saude-tabnet/) (Figure 1). DATASUS provides publicly available data that support objective analyses of the health situation, evidence‐based decision‐making, and the development of health action programs.

Exploratory statistical techniques were employed for data analysis, enabling a clearer visualization of the general characteristics of the data. The analyses were categorized by procedure type, period (month/year), across different regions of Brazil.

The initial data were presented in tables showing the total annual number of cases for each procedure by region and for Brazil. Due to the milestones of the pandemic, the years were considered to start in March and end in February of the following year. The variation representing an increase or decrease was calculated based on the comparison between the base year and the previous year.

A second analysis was conducted using monthly data from the analyzed periods, which were divided as follows: pre‐pandemic period (January 2018 to February 2020), lockdown period (March 2020 to December 2020), vaccination period (January 2021 to March 2022), and post‐pandemic period (April 2022 to June 2024). To compare the evaluated periods, the ANOVA test was used, along with pairwise multiple comparisons by Bonferroni.

Statistical analysis was performed using the SPSS software version 25.0, and the significance level for the tests was set at 5%.

Heatmap charts were created showing the monthly average number of corneal transplants by region and period. Each cell represents the average number of transplants in a specific region and period, with the color intensity indicating the quantity. Regions with lower numbers appear in lighter shades, while regions with higher transplant numbers are represented in darker shades.

Annual trends in corneal transplantation were first evaluated based on absolute yearly numbers and year‐to‐year percentage variation across regions. Complementary analyses using average monthly data were subsequently performed to assess differences between the pre‐pandemic, lockdown, vaccination, and post‐pandemic periods.

Finally, Statistical analyses and reporting were conducted in accordance with established recommendations for clinical research, including the SAMPL guidelines [27] and the statistical reporting guidelines proposed by Assel et al. [28]. Comparisons of the average monthly number of corneal transplants across periods were pre‐specified and performed using one‐way analysis of variance (ANOVA). When a significant overall difference was identified, pairwise comparisons were conducted with Bonferroni adjustment for multiple testing. Annual percentage variations were calculated descriptively to characterize year‐to‐year changes and were analyzed separately from the period‐based comparisons. No exploratory or post hoc subgroup analyses beyond the predefined regional and temporal stratifications were performed. All statistical tests were two‐sided, and a significance level of 5% (*p* < 0.05) was adopted. Statistical analyses were performed using IBM SPSS Statistics software, version 25.0 (IBM Corp., Armonk, NY, USA).

## 3 Results

During the pandemic year (2020), the number of corneal transplants decreased significantly across the five Brazilian regions (North, Northeast, Southeast, South, and Midwest). There was a 54% drop in the number of transplants compared to 2019. The regions most affected were the South (−65%), Northeast (−56%), and Southeast (−53%). In 2021, with the progress of vaccination, there was a visible recovery in the number of transplants. The national total number increased by 86% compared to 2020. The most significant recoveries occurred in the South (121%), Northeast (106%), and Southeast (86%). The number of transplants continued to grow in 2022, with an 18% increase in the total number of transplants in Brazil compared to 2021. All regions showed an increase in the number of surgeries, with the North (143%) and South (36%) seeing the largest changes. In 2023, there was a slight decrease of 5% in the total number of transplants in Brazil. The North had the largest drop (−29%), followed by the Southeast (−7%) and the Midwest (−3%). Only the South (4%) and Northeast (1%) did not experience a decrease (Table 1).

**Table 1:** Number of corneal transplant cases by Brazilian region from 2018 to 2023 and the variation compared to the previous year.

Períod	Year*	Number of corneal transplantations						Variation compared to the previous year					
		North	Northeast	Southeast	South	Midwest	Brazil (total)	% North	% Northeast	% Southeast	% South	% Midwest	% Brazil
Pre‐pandemic	2018	492	2.165	3.957	1.032	893	8.539						
Pre‐pandemic	2019	428	2.030	4.143	1.206	726	8.533	−13%	−6%	5%	17%	−19%	0,1%
Lockdown	2020	203	889	1.952	427	450	3.921	−53%	−56%	−53%	−65%	−38%	−54%
Vaccination	2021	191	1.835	3.635	945	668	7.274	−6%	106%	86%	121%	48%	86%
Post‐pandemic	2022	464	1.953	4.090	1.288	806	8.601	143%	6%	13%	36%	21%	18%
Post‐pandemic	2023	330	1.970	3.788	1.341	780	8.209	−29%	1%	−7%	4%	−3%	−5%

**Figure 2:**
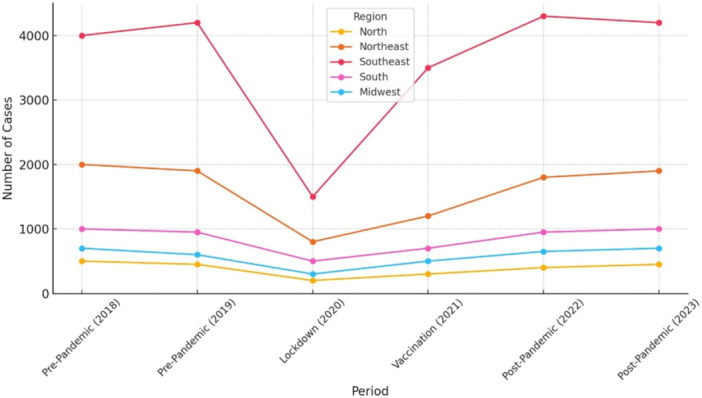
Evolution of the number of corneal transplants across the five Brazilian regions (North, Northeast, Southeast, South, and Midwest) between 2018 and 2023.

All regions experienced a significant drop in the number of transplants in 2020, due to the impacts of the COVID‐19 pandemic. The Southeast region stood out with the highest number of corneal transplants. From 2018 to 2019, there was a slight decrease in the number of cases, followed by a substantial decline in 2020. However, from 2021 onward, there was a strong recovery, with the number of transplants reaching nearly 4000 in 2023. The Northeast showed a gradual decrease in the number of transplants until 2020, when the number of cases hit its lowest point. From 2021, there was a steady increase, stabilizing at around 2000 transplants in the subsequent years. In the South, corneal transplant activity increased by 17% from 2018 to 2019, before experiencing a significant reduction in 2020. Starting in 2021, there was a gradual recovery, though the numbers stayed below 2000. The Midwest also followed a downward trend until 2020 but experienced a slight recovery starting in 2021, stabilizing below 1000 cases. The North region showed the lowest number of corneal transplants throughout the entire period. As with the other regions, there was a decline in 2020, but the number of cases remained consistently low, staying below 500 (Table 1 and Figure 2).

**Figure 3:**
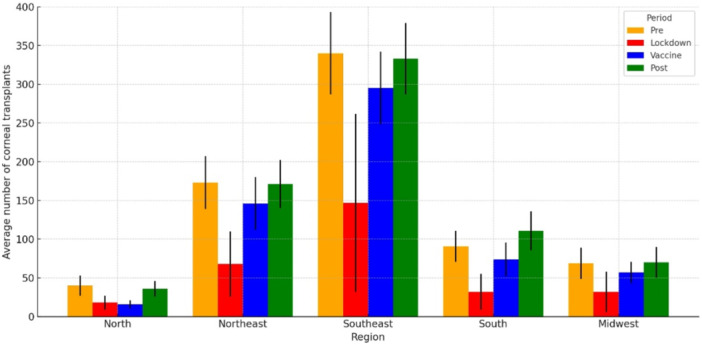
Average number of corneal transplants in the Brazilian regions across the pre‐pandemic, lockdown, vaccine, and post‐pandemic periods.

The analysis of the average monthly number of corneal transplants performed across the five regions of Brazil and the four evaluated periods, revealed a statistically significant difference among the periods in all regions studied (*p *< 0.001). Nationally, the average number of corneal transplants dropped by 41.7% during the Lockdown compared to the pre‐pandemic period, from an average of 713 (± 79) to 297 (± 201). During the vaccination period, the average increased significantly to 589 (± 93), reflecting consistent progress in the recovery of transplants. In the post‐pandemic period, the national average was 721 (± 82), slightly above the Pre‐pandemic level, suggesting consistent recovery at the national level (Table 2 and Figure 3).

**Table 2:** Data on the average monthly number of corneal transplants performed across the five regions of Brazil, distributed over the four evaluated periods.

Region	Pre‐pandemic	Lockdown	Vaccination	Post‐pandemic	*p* value*	Reduction during lockdown (%)
North	40 (13)	18 (9)	16 (5)	36 (10)	< 0.001	55.0
Northeast	173 (34)	68 (42)	146 (34)	171 (31)	< 0.001	60.7
Southeast	340 (53)	147 (115)	295 (47)	333 (46)	< 0.001	56.8
South	91 (20)	32 (23)	74 (22)	111 (25)	< 0.001	64.8
Midwest	69 (20)	32 (26)	57 (14)	70 (20)	< 0.001	53.6
Brazil	713 (79)	297 (201)	589 (93)	721 (82)	< 0.001	58.3

*Note:* Values are presented as the average monthly number of corneal transplants, with standard deviation shown in parentheses. Data are distributed according to the pre‐pandemic (January 2018 to February 2020), lockdown (March 2020 to December 2020), vaccination (January 2021 to March 2022), and post‐pandemic (April 2022 to June 2024) periods. Percentage reduction was calculated as the relative decrease in the average monthly number of corneal transplants during the lockdown period compared with the pre‐pandemic period, using the pre‐pandemic average as the reference.

* *p* values were obtained using one‐way analysis of variance (ANOVA) with multiple comparisons adjusted by the Bonferroni method. North: *p* < 0.05 for pre vs. lockdown and vaccination; lockdown vs. post; vaccination vs. post. Northeast: *p* < 0.05 for pre vs. lockdown; lockdown vs. post; vaccination vs. post. Southeast: *p* < 0.05 for pre vs. lockdown; lockdown vs. vaccination and post; vaccination vs. post. South: *p* < 0.05 for pre vs. lockdown and post; lockdown vs. vaccination and post; vaccination vs. post. Midwest: *p* < 0.05 for pre vs. lockdown; lockdown vs. vaccination and post; vaccination vs. post. Brazil: *p* < 0.05 for pre vs. lockdown and vaccination; lockdown vs. vaccination and post; vaccination vs. post.

During the lockdown period, all Brazilian regions experienced a marked reduction in the average number of corneal transplants compared with the pre‐pandemic period. The reduction reached 55.0% in the North, 60.7% in the Northeast, 56.8% in the Southeast, 64.8% in the South, and 53.6% in the Midwest. At the national level, the decrease was 58.3%. These differences were statistically significant across regions (ANOVA with Bonferroni correction, *p*< 0.001). Recovery began in the vaccination period, with notable increases in transplant numbers across regions, except by the North region that showed a non‐significant reduction compared to the lockdown. During the post‐pandemic period, the Northeast and Southeast regions approached pre‐pandemic levels, while the Midwest did not fully recover to baseline values. Statistically significant differences were observed across all periods (pre, lockdown, vaccine, post), highlighting the impact of the pandemic and the subsequent recovery efforts (Table 2 and Figure 3).

**Figure 4:**
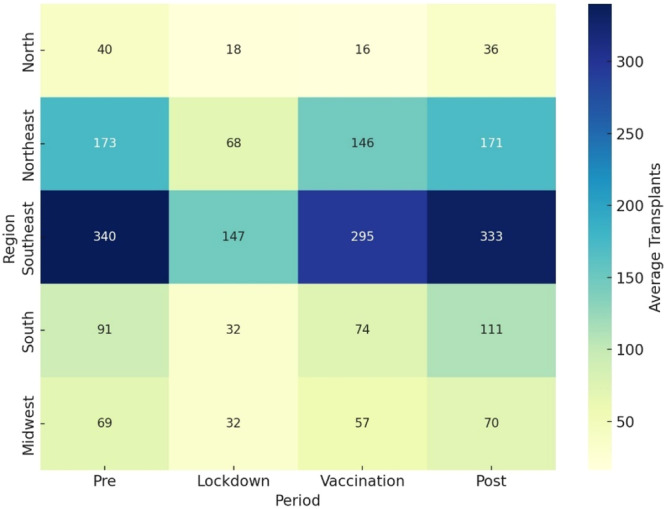
Heatmap of the average monthly number of corneal transplants by region and period. Each cell represents the average number of transplants in each region and period, with the color intensity indicating the quantity. Regions with lower numbers appear in lighter shades, while regions with a higher number of transplants are darker.

A heatmap was created to visualize the impact of the lockdown period and the recovery over the subsequent periods across different regions. It illustrates the differences between the evaluated periods in terms of the average number of corneal transplants in each region, with a focus on comparisons relative to the lockdown period (Figure 4).

## 4 Discussion

Beginning in March 2020, when COVID‐19 was declared a global pandemic, the rapid spread of SARS‐CoV‐2 posed a severe challenge to healthcare systems in Brazil and worldwide. This impact was felt in the field of corneal transplants. The pandemic led to significant disruptions in donor identification and screening processes, as well as a broader reorganization of healthcare services, which severely affected the availability of organs and tissues for transplantation [29, 30].

From 2017 to 2019, Brazilian Eye Banks obtained an annual average of 16,850 donors with 31,791 processed corneas, and 17,205 corneal transplantations, according to the Tissue Banks Production Data Evaluation Reports [31]. Data from 37 Brazilian eye banks on corneal donations and transplants in the country, from January 2020 to August 2020, indicated an 85.7% decrease in corneal donations in Brazil when comparing the first 2 months of 2020, before lockdown, to the initial 2‐month period, during the lockdown [19]. Our data showed a 56% decrease in the Brazilian cornea transplant during the lockdown year compared to the previous pre‐pandemic year.

A significant decline has also been evaluated and observed worldwide corroborating with our findings, as described below. The report from the Italian National Eye Bank, which analyzed data from all 13 eye banks, showed a statistically significant decrease in the number of donor corneas recovered in 2020 compared to a similar period in 2019 and 2018, resulting in a significant reduction in corneal transplants during the COVID‐19 period [20, 21]. A study conducted in Turkey, found not only a sharp decline in transplants in 2020, but also a notable increase in post‐surgical complication rates, which could be attributed to delayed diagnosis of corneal rejections due to impaired post‐operative follow‐up regimens [22]. In India, 8 out of the 20 existing eye banks in the country suspended their corneal preservation between April and June 2020. Additionally, two‐thirds of surgeons (66.1%, *n* = 41/62) did not perform any corneal transplants during the same period, resulting in a significant drop in corneal tissue processing and utilization during the lockdown [23]. Finally, data from 64 eye banks in 26 European countries, indicated a decrease in corneal tissue preservation of 38%, 68%, and 41%, respectively, in March, April, and May 2020, compared to the average of the previous 2 years, while grafts decreased by 28%, 68%, and 56%, corresponding to 3866 untreated patients over the course of 3 months [24].

It is worth mentioning that modern lamellar corneal transplant for endothelial decompensation (Endothelial Keratoplasty–EK) requires a reasonable visualization of the anterior chamber. The more wide‐spread techniques for EK are Descemet Stripping Automated Endothelial Keratoplasty (DSAEK) and Descemet Membrane Endothelial Keratoplasty (DMEK). They allow a much safer surgery with significantly improved results as compared to the traditional penetrating keratoplasty [32]. Delay in providing surgical treatment for these patients may prevent the use EK and may require a full‐thickness corneal graft with an obvious decrease in visual prognosis.

In Brazil, the implementation of social distancing measures was first announced by the Federal District at the beginning of the pandemic. Each state adopted social distancing measures according to its reality and at different times [4, 5]. In our study, we observed that variations over the years reveal regional differences in corneal transplant procedures, likely influenced by factors such as healthcare infrastructure, donor availability, and local health policies. To place the findings of the present study within the Brazilian context, it is necessary to recognize the substantial heterogeneity in corneal transplantation capacity among states and regions. Data from the Brazilian Association of Organ Transplantation (ABTO) [33] indicate wide variation in waiting list size at the end of 2024, ranging from more than 5600 patients in São Paulo (a Southeast state) to fewer than 100 patients in Ceará (a Northeast state). States in the Southeast region, particularly São Paulo, Rio de Janeiro, and Minas Gerais, accounted for the largest absolute waiting lists. However, absolute waiting list size does not adequately reflect system performance. In 2024, São Paulo performed 5,584 corneal transplants, corresponding to 99.3 percent of its waiting list, whereas Rio de Janeiro and Minas Gerais performed 611 and 995 procedures, respectively, addressing only a limited proportion of their estimated demand. When annual demand estimates are considered, only a small number of states were able to meet or exceed projected needs, including Ceará, the Federal District, and São Paulo. These differences are associated with the unequal distribution of transplant teams nationwide. The Southeast region concentrates more than half of the registered corneal transplant teams in the country, while some states in the North have no registered teams. Although these structural disparities preceded the COVID‐19 pandemic and are not the main focus of the present analysis, they provide relevant context for interpreting the magnitude of service disruption and the variability in post‐pandemic recovery observed among regions.

All regions experienced a significant drop in the number of transplants in 2020, due to the impacts of the COVID‐19 pandemic, which affected medical procedures and elective surgeries. From 2021, there was a recovery across all regions, although the intensity of this recovery varied. The Southeast led both in total numbers and in the pace of recovery. Nevertheless, the Northeast and South regions showed marked year‐to‐year increases in transplant activity after 2021, as reflected in the annual trends presented in Table 1. The North and Midwest regions had the lowest number of transplants, suggesting a regional disparity in access to or demand for this type of procedure.

Garcia et al. [19] reported that the pandemic consequence would be a 14.7% increase in the number of patients waiting for corneal transplants, rising from 12,000 patients on January 31, 2020, to 14,000 patients on August 31, 2020. Overall, our data indicate that although all regions experienced significant drops in the average monthly number of corneal transplants during the lockdown, there was a recovery in subsequent periods, especially after the start of vaccination. Most regions reached or approached pre‐pandemic levels in the post‐pandemic period, with some regions even exceeding these averages, as seen in the South and at a national level, which may suggest efforts to compensate for delayed procedures. Despite regional differences, Brazil managed to promote a recovery in corneal transplants after the initial impact of the pandemic. Moriyama et al [34] estimated in May 2021 that the monthly corneal transplant rates would need to increase by approximately 34% in the state of São Paulo and 91% in Brazil for the corneal transplant waiting list to return to pre‐pandemic levels within the next 2 years. However, even with the recovery in the number of transplants, our data indicates that the number of transplants has not been fully compensated for after the pandemic, and this imbalance has been further exacerbated by the pandemic.

Even before the COVID‐19 pandemic, there was a significant imbalance between the supply and demand for corneal tissue, with an estimated of at least 53.3% of the global population lacked access to corneal tissue [15]. Despite efforts to recover corneal transplantation, as seen in the present study, there are limitations, likely due to the scarcity of available corneas and a shortage of professionals. This makes it difficult to address the backlog of demand, suggesting that the impact of events like the pandemic could be long‐lasting.

The long‐term effects of national recommendations in response to the pandemic on the visual health of the Brazilian population and worldwide require continued analysis. The adaptation of health services to the new scenario, combined with the collection of accurate data on the distribution and execution of surgeries, will be essential to ensure that ophthalmological care not only returns to previous levels but also becomes more resilient to future crises. Thus, mitigating the negative impacts of the pandemic becomes a priority to ensure universal and quality access to eye health in Brazil. Studies like this demonstrate the importance of quantifying trends in ophthalmic procedures in the retrospective evaluation of surgical interruptions and in the prospective accommodation of delayed surgeries.

## Author Contributions

**Josue Geraldo Lessa:** conceptualization, data curation, methodology, writing – original draft. **Amélia Soares de Melo:** data curation, investigation, writing – original draft. **Leandro Vassuler Baldon:** data curation, writing – original draft. **Bernardo G. L. Carvalho:** data curation, investigation. **Nathalia Sernizon Guimaraes:** conceptualization, investigation, methodology. **Aleida N. Soares:** formal analysis, methodology, validation, writing – original draft. **Bruno Lovaglio Cançado Trindade:** conceptualization, methodology, project administration, resources, supervision, writing – original draft, writing – review and editing. **Fernanda Ludolf:** conceptualization, formal analysis, funding acquisition, project administration, supervision, validation, writing – original draft, writing – review and editing.

## Ethics Statement

The authors have nothing to report.

## Consent

The authors have nothing to report.

## Conflicts of Interest

The authors declare no conflicts of interest.

## Transparency Statement

The lead author, Fernanda Ludolf, affirms that this manuscript is an honest, accurate, and transparent account of the study being reported; that no important aspects of the study have been omitted; and that any discrepancies from the study as planned (and, if relevant, registered) have been explained.

## Data Availability

The data supporting the findings of this study are publicly available from the Brazilian Health Information System (DATASUS) through the TABNET platform (https://datasus.saude.gov.br/informacoes-de-saude-tabnet/). All data used in the analyses were obtained from this open‐access database.
